# The Development and Initial Psychometric Testing of a Patient-Reported Outcome Measure to Assess COPD-Related Emotional Distress

**DOI:** 10.2147/COPD.S585311

**Published:** 2026-05-08

**Authors:** Gabriela Schmid-Mohler, Christine Hübsch, Marianne Mueller, Rafael Imhof, Katja-Daniela Jordan, Christian Clarenbach, Gian-Marco Monsch, Janelle Yorke

**Affiliations:** 1 Centre of Clinical Nursing Science, University Hospital Zurich, Zurich, Switzerland; 2 Division of Pulmonology, University Hospital Zurich, Zurich, Switzerland; 3 School of Health Professions, Bern University of Applied Sciences, Bern, Switzerland; 4 Nursing Department C, University Hospital Zurich, Zurich, Switzerland; 5 Department of Consultation-Liaison Psychiatry and Psychosomatic Medicine, Zurich, Switzerland; 6 Faculty of Medicine, University of Zurich, Zurich, Switzerland; 7 Department of Thoracic Surgery, University Hospital Zurich, Zurich, Switzerland; 8 School of Nursing, The Hong Kong Polytechnic University, Kowloon, Hong Kong; 9 Division of Nursing, Midwifery and Social Work, The University of Manchester, Manchester, UK

**Keywords:** pulmonary disease chronic obstructive, psychological distress, patient-reported outcome measures, psychometrics, depression, anxiety, self-management

## Abstract

**Purpose:**

Emotional distress is prevalent in patients with COPD and highly relevant to patients. Research in chronic disease indicates that illness-related emotional distress might be specific and sensitive enough to explain self-management behaviour. However, as no currently available instrument assesses COPD-related emotional distress (CRED), it has only been assessed regarding overall distress, using mainly anxiety and depression as outcomes. Therefore, this study aimed to develop and test a preliminary item list to measure CRED.

**Patients and Methods:**

Following Food and Drug Administration (FDA) guidelines, a multistep mixed-method study was conducted. Based on an earlier qualitative study and literature review, a conceptual framework and item list were developed. The item list’s content validity was assessed via patient interviews using cognitive debriefing techniques and a survey involving a panel of clinicians. Finally, its psychometric properties were tested in a cross-sectional study. Construct validity was established by comparing the CRED-V1 with established questionnaires like the COPD Assessment Test (CAT), the Hospital Anxiety and Depression Scale (HADS), and the modified Medical Research Counsel (mMRC) dyspnoea scale.

**Results:**

The first German COPD-Related Emotional Distress questionnaire–version 1 (CRED–V1) contained 36 items. Its content validity was confirmed by nine patients and ten clinicians. Psychometric testing in 264 patients with COPD revealed two formative (symptom- and treatment-related) and four reflective (restricted mobility-, restricted relationship-, disease unpredictability- and stigma-related) subcategories. For all reflective subscales, Cronbach’s alpha values were >0.8. Structural equation modelling was possible for 32 items: An R^2^ value of 0.656 allowed the calculation of a CRED total score (CRED–TS). A regression model using the CRED-TS as the outcome variable showed that the most important explanatory variables were the CAT and HADS depression scores.

**Conclusion:**

This work reports the initial development of a new innovative tool for the assessment of CRED in patients with COPD.

## Introduction

Patient-reported outcome measures (PROMs) assess and track patient-concerning concepts, and assess the effectiveness of interventions. One prerequisite for a PROM is that it must assess a concept that matters to patients.^1^ In Chronic Obstructive Pulmonary Disease (COPD), emotional distress is one such concept: patients report repeatedly how their symptoms significantly limit their activities and affect their daily lives, leading to emotional distress.^2–6^

**Figure 1:**
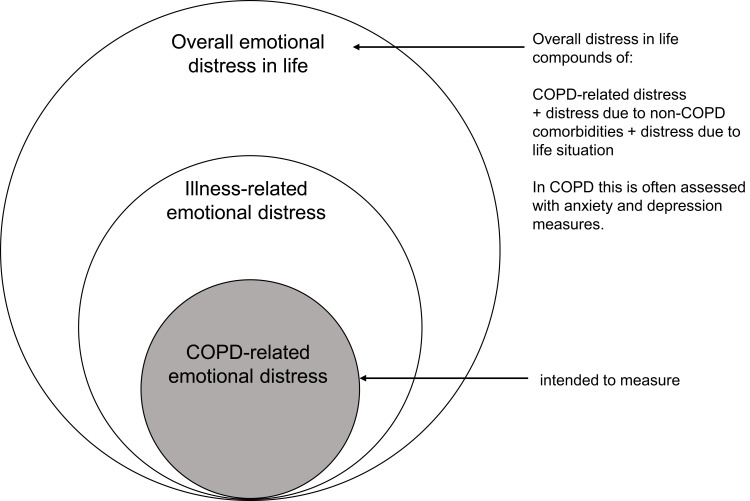
Concept: illness-related emotional distress in COPD (CRED).

In previous work, emotional distress was defined as “the level of perceived burden experienced by patients due to symptoms, treatments, restrictions in life roles, and the unpredictability of the condition”, ie., the extent to which their illness-related burden leads to feelings such as sadness, anger, shame and anxiety.^7^ Patients with COPD report six main sources of COPD-related burden: symptoms, treatments, physical mobility restrictions, limitations to social participation, perceived stigma, and the unpredictability of COPD’s course.^8^ Several also note non-COPD sources such as comorbidities, life events and their overall living situation that contribute to emotional distress. A well-formulated PROM is crucial to distinguish COPD-related emotional distress from that arising from comorbidities and overall life-related distress (Figure 1).

To our knowledge, the COPD-Anxiety Questionnaire (CAF) is the one instrument so far that assesses a form of COPD-specific emotional distress.^9^ It focuses on themes connected with anxiety and does not refer to other topics connected with other burdensome emotions, such as sadness, anger, and shame. Lacking a purpose-developed instrument to measure COPD-related emotional distress (CRED), studies aiming to assess these commonly applied measures of global depression and anxiety. Eg., when a recent review examined the association of emotional distress with adverse outcomes in patients with COPD, 18 of 19 studies predominately focussed on depression.^10^ The non-condition-specific instruments most commonly used to report on emotional distress are the Hospital Anxiety and Depression Scale (HADS), the Center for Epidemiologic Studies–Depression scale (CES–D), the Patient Health Questionnaire 9 (PHQ–9), and the Beck Depression Inventory (BDI).^10–12^ These non-condition-specific measures are not able to distinguish between distress caused by COPD itself and that caused by comorbidities.

To monitor emotional distress, condition-specific PROMs offer two main advantages: They lower the risk of over-reporting anxiety and depression,^13^ and measure condition-related emotional distress, which is likely to be more specific and sensitive than general approaches. The latter principle has been well-illustrated in diabetes populations, where researchers tracking diabetes-related emotional distress have linked it with suboptimal self-management, such as poorer blood sugar control.^14^,^15^ In patients with COPD, higher COPD-related anxiety, as measured by the revised CAF, was associated with poorer rehabilitation outcomes, such as lower functional exercise capacity and lower health-related quality of life.^16^ One possible explanation is that patients who lack key self-management skills cannot achieve a sense of (new) normality,^17^ leading to feelings of powerlessness. In various chronic illnesses, disempowerment has been associated with chronic grief and depression.^18^,^19^ Such illness-related distress commonly responds to psychoeducational interventions, including mindfulness-acceptance approaches,^20^,^21^ but not to education-free psychological interventions.^22^

The objective of this study was to develop a disease-specific PROM to measure CRED and to evaluate its psychometric properties. The questionnaire is based on our earlier definition of COPD-related emotional distress: the level of perceived burden due to symptoms, treatments, restrictions in life roles, and the unpredictability of COPD regarding symptoms and their severity.^7^

## Methods

**Figure 2:**
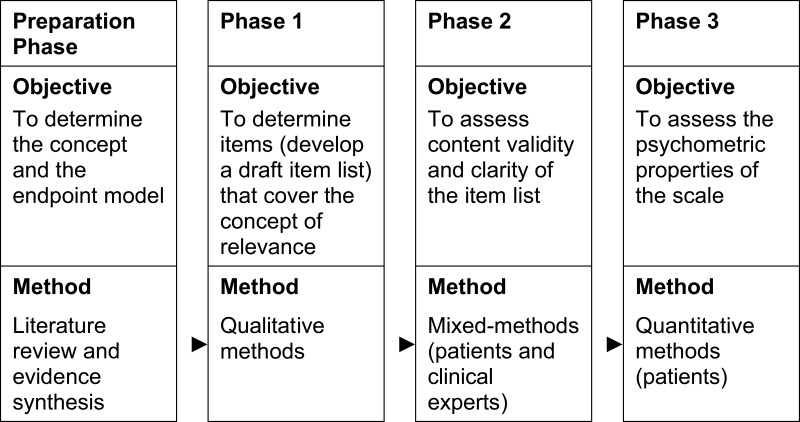
Overview of aims and methods, based on FDA guideline.

Following the FDA guidelines for PROM development and validation,^23^,^24^ qualitative (Phase 1), mixed (Phase 2) and quantitative methods (Phase 3) were applied to develop the German CRED-V1. An overview of aims and methods is illustrated in Figure 2. The study complied with the Declaration of Helsinki.

### Phase 1: Item Identification

#### Design

Items were identified via qualitative methods. The conceptual framework for this PROM’s development was derived from the model of emotional distress in respiratory diseases, previously published by our team.^7^ This provided the four main symptom-related categories: symptoms, treatment, daily life-role restrictions and unpredictability. Based on that previous qualitative work, items were then generated for each category. For validation purposes, the conceptual framework comprises three levels, with items defined for each level. Level 1 assesses general COPD-related emotional distress; level 2 assesses distress relating to one of the previous mentioned subcategories, eg. treatment; and level 3 assesses distress relating to a specific source, eg. oxygen therapy. Items at level 3 that are subordinate to a subcategory (eg. treatment) should explain the variance of the corresponding level-2-item.

#### Data Sources

Two data sources were used, both previously published by our team:

- Transcripts of one-to-one interviews conducted September 2018–May 2019 for a study exploring sources of emotional distress in patients with COPD at University Hospital Zurich, Switzerland.^8^
- Patient quotes extracted from a systematic literature review of qualitative studies in February 2018, repeated on August 2019 exploring patients’ COPD exacerbation experiences.^25^

#### Analysis

Framework analyses were conducted on both sources.^26^ For each item’s wording, all related patients’ narratives were considered alongside those patients’ characteristics, eg., disease stage.

### Phase 2: Content Validity and Item List Clarity

#### Design

From June to August 2019, a convergent mixed-method design test was conducted to determine all proposed items’ content validity and clarity. Clinicians’ (quantitative) and patients’ (qualitative) data were collected simultaneously, analysed separately, and synthesized. Where necessary, items were adapted. Because no health-related data were collected, the Canton of Zurich Ethics Committee confirmed that the project fell outside their responsibility (KEK Nr. Req-2019-00415).

#### Sample and Setting

##### Clinicians

Health professionals (physicians, nurses, physiotherapists, pastoral caregiver) from Switzerland with expertise in the care of patients with COPD were included.

##### Patients

Inclusion criteria included a confirmed COPD diagnosis, ongoing treatment at University Hospital Zurich and an age ≥18. Exclusion criteria were inability to speak and read German.

#### Data Collection

Experts were contacted by email. Patients treated at University Hospital Zurich were informed. After providing written informed consent, they were interviewed in a face-to-face contact.

#### Variables

##### Clinicians

Using a self-developed survey, each of the 36 items’ relevance, designation to category and clarity were assessed. Relevance and clarity were assessed on a 4-point Likert Scale (1 = not relevant/clear, 2 = somewhat relevant/clear, 3 = relevant/clear, 4 = very relevant/clear). Category designations (symptom, treatment, restriction in daily life, or unpredictability) were assessed nominally.

##### Patients

One-to-one cognitive debriefing interviews were conducted. For each item, patients’ perspectives were explored regarding relevance, content coverage and clarity.^27^

#### Analysis

##### Clinicians

Each item/question’s relevance, clarity and category designation were analysed by calculating the percentage of panellists who responded favourably (3 = relevant/clear, 4 = very relevant/clear) or marked the correct category. Each item’s total score (100%) was the number of panellists who provided any answer for it. Scores ≥80% were rated “good”. Items scoring <80% were revised or deleted.^28^ If panellists noted that an item exceeded the concept, or that areas of content were inadequately covered, the supervisory team (first and second author) reviewed it.

##### Patients

Interviews were audio-recorded, transcribed and analysed deductively using framework analysis^29^ with a pre-determined index. This followed the questionnaire format (introduction and items) and assessment criteria both for the overall questionnaire and for each item (eg. relevance, coverage of content, clarity). Items with responses indicating that they were always/sometimes relevant or had been relevant in the past (either for the respondent or for other patients), were considered relevant. The total number of relevant answers was divided by the number of interviewed patients who provided any relevance-related statement. If the quotient was ≥80%, the question was considered relevant. Regarding content coverage and language clarity, responses reporting either directly or indirectly (via discrepancies between developer-intended and patient-reported content), that items were not clear or not well-covered were considered, respectively, unclear or not well-covered.

#### Synthesis

Clinicians’ and patients’ views were summarised in a matrix. Their views were compared for each item, considering its relevance, content coverage and clarity. Any potential adaptations were discussed with a supervisory team.

### Phase 3: Psychometric Testing of the Scale

#### Design

A single-centre cross-sectional study was conducted (April 2020– January 2023), approved by the Canton of Zurich Ethics Committee (KEK-Nr. 2020–00355).

#### Sample and Setting

Patients who had been treated (as in- or outpatients) at University Hospital Zurich over the past 24 months, had a confirmed COPD diagnosis, and were aged ≥18 were included. Patients unable either to speak or read German, or had a diagnosis of dementia or delirium were excluded.

#### Data Collection

Each eligible patient was mailed an information package including the questionnaires and consent form. The study nurse contacted them one week later, informed them orally about the study, answered questions and checked for exclusion criteria. Those agreeing to participate signed and returned a written informed consent with their completed questionnaire in a preaddressed, pre-stamped envelope. Where data were omitted, patients were contacted by the study nurse.

#### Variables

Demographic (age, sex, civil status) and clinical data (GOLD grade, FEV_1%predicted_, use of oxygen, body mass index) were retrieved from the electronic patient chart.

On a 6-point Likert scale (0 = none, 5 = extreme), the “CRED–V1” assessed emotional distress regarding 36 distress sources. “Situation does not apply to me” (no distress at all) was scored as “0” as this indicates an absence of distress regarding this topic.

To test construct validity, three pencil/paper questionnaires were used: the 8-item COPD Assessment Test (CAT), quantifying COPD’s impact on daily life and wellbeing (6-point Likert scales; sum score, 0 = no impact, 40 = maximal impact);^30^ the single-item Modified Medical Research Council (mMRC) dyspnoea scale, assessing activity limitations due to dyspnoea (5-point Guttman scale; 0 = no impact, 4 = maximum impact);^31^ and the 14-item Hospital Anxiety and Depression Scale (HADS) measuring depression and anxiety (two 7-item subscales using 4-point Likert scales (0 = no symptoms; 21 = maximum symptoms per subscale).^32^

#### Analysis

The following statistical methods were used. 1) Descriptive statistics clarified items’ relevance and answer categories’ distributions. 2) Exploratory analysis helped identify items’ factor structure (dimensionality). “Treatment” and “symptoms” were approached as formative constructs, and “unpredictability” and “restriction in daily life” as reflective constructs. For the latter, exploratory factor analysis with Varimax rotation was used. 3) To test a comprehensive theoretical CRED model that would allow the calculation of a weighted CRED total score (CRED–TS), the partial least squares approach to structural equation modelling (PLS–SEM) was chosen. Items of all three levels were included in this model. 4) To assess the CRED–V1’s construct validity against other questionnaires (HADS, CAT, mMRC), multiple regression was used. A model was developed using the CRED–TS as outcome variable and the HADS, CAT, and mMRC dyspnoea scores, FEV1%predicted, age and sex as predictor variables. Data sets with missing CRED–V1 values were excluded list-wise. P-values ≤0.05 were considered statistically significant. Weights’ and loadings’ significances were determined via one-tailed tests. Statistical software packages used were IBM SPSS Statistics 29 and R 4.3.3 with the plspm package.

## Results

### Phase 1: Item Identification

#### Sample Characteristics

##### Source A

Eleven patients (median age 67, 7 female) participated (3 COPD GOLD stage I; 2 stage II; 3 stage III; 3 stage IV). Five were long-term oxygen users. For further details, see Zanolari, Handler-Schuster, Clarenbach and Schmid-Mohler.^8^

##### Source B

Fifteen qualitative international studies (n = 326 patients with COPD; 140 female; 2012–2017) were reviewed.^25^

#### Item List

**Figure 3:**
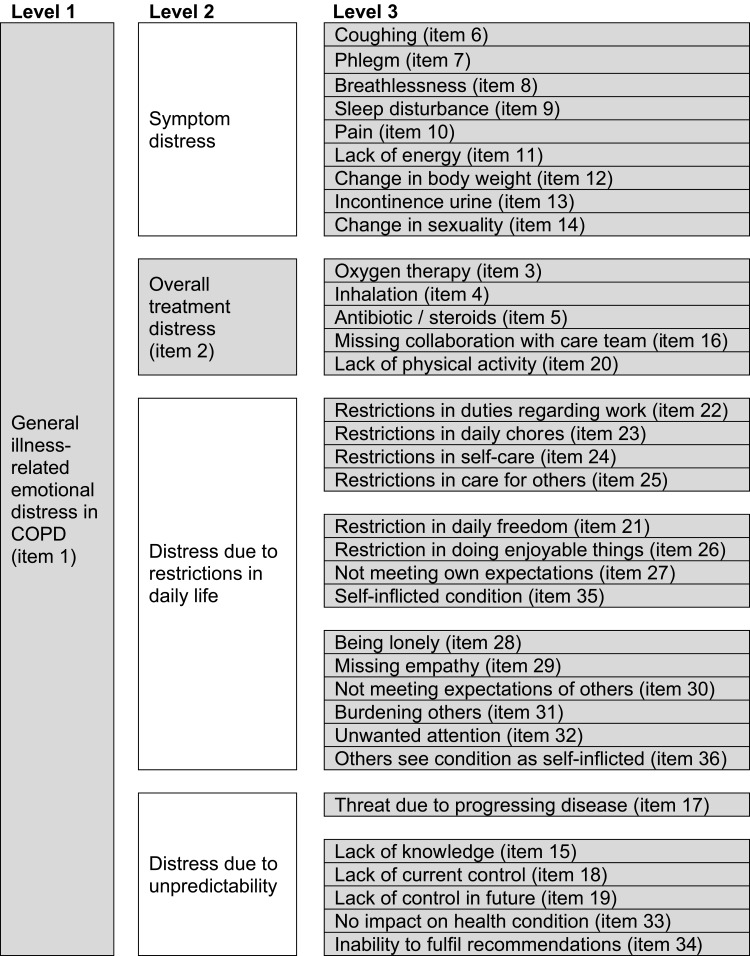
The conceptual framework of CRED.

Thirty-four distress sources identified in the four main categories (symptoms, treatment, restrictions in daily life and unpredictability) are listed in Figure 3, column “Level 3”.

For validation purposes, two items were added: “general illness-related distress” (Item 1, level 1) and “overall treatment distress” (Item 2, level 2). “Overall treatment distress” (Item 2) reflects all level 3 treatment items (Items 3–5, 16, 20). Additionally, a “situation does not apply to me” checkbox was added to each item.

### Phase 2: Content Validity and Item List Clarity

#### Sample Characteristics

Ten clinicians (4 nurses, 3 pulmonologists, 2 physiotherapists, 1 pastoral caregiver; median experience: 14 years (range 3–30)) and nine patients (5 female; median age 69 (range 52–81); median FEV_1 predicted_ 35% (range 17–58%)) participated. Mean duration of patients’ interviews was 54 minutes (range 31–75).

#### Ratings of Relevance, Content Allocation and Clarity by Clinicians and Patient

##### Overall Impression

All clinicians rated the “emotional distress” concept as relevant and comprehensively covered by the items. The introduction’s layout and wording were clear.

##### Clinicians Assessed the Scoring as Suitable

Two experts suggested a timeframe of 1–4 weeks. Two found the questionnaire rather complex and suggested deleting items. However, patients found it clear and easy.

##### Item Relevance

Of the 36 original items, clinicians or patients rated 32 relevant. Both groups questioned four items’ relevance: “antibiotics/steroids” (Item 5), “missing empathy” (Item 29), “self-inflicted condition” (Item 35), and “others see condition as self-conflicted” (Item 36). At this step, no item was deleted. However, when patients noted that “restriction in duties regarding work” (Item 22) did not apply to retirees (most patients), this item was adjusted to read “work/volunteering”.

##### Items’ Clarity

From the clinicians’ perspective, all items’ wordings were clear. Patients reported problems with the term “incontinence urine” (Item 13), which was rephrased.

##### Item-Level Content Coverage by Patients

Contrasting patient reports against the intended meaning exposed three problematic items: “treatment distress” (Item 2), “lack of physical activity” (Item 20) and “not able to fulfil recommendations” (Item 34). The latter two were adapted.

##### Clinicians’ Item-Category Allocations

As no category fit “lack of physical activity” (Item 20), “activity” was replaced with “training”.

For individual item ratings, see Table S1.

### Phase 3: Psychometric Testing of the Scale

#### Sample Characteristics

This cross-sectional study included 264 patients (mean age: 69.4 (SD 8.8); 150 (56.8%) male; mean BMI 24.9 (SD 5.2); mean FEV^1^, % predicted 48.9 (SD 22.0); oxygen use during study period 88 (33.3%); mean CAT score 18 (SD 7.1); mean mMRC dyspnoea score 1.9 (SD 1.1) (Table 1).

**Table 1:** Sample Characteristics and Questionnaire Results (n=264)

Variable	
Age, mean [SD]	69.4 [8.8]
Nationality, n (%)	
Swiss	241 (91.3%)
Other	22 (8.4%)
Missing	1 (0.4%)
Civil status, n (%)	
Single	45 (17%)
Married	108 (40.9%)
Divorced	58 (22%)
Widowed	31 (11.7%)
Separated	5 (1.9%)
Missing	17 (6.4%)
COPD GOLD, n (%)	
1	29 (11%)
2	81 (30.7%)
3	82 (31.1%)
4	58 (22%)
Missing	14 (5.3%)
FEV1 predicted %, mean [SD] (n=250)	48.9 [22]
Long-term oxygen at home, n (%)	
Yes	88 (33.5%)
No	167 (63.3%)
Missing	9 (3.4%)
BMI, mean [SD] (n=253)	24.9 [5.2]
CAT, mean [SD] (n=255)	18.0 [7.1]
mMRC, n (%)	
0	26 (9.8%)
1	66 (25.0%)
2	90 (34.1%)
3	49 (18.9%)
4	18 (6.8%)
Missing	15 (5.7%)
HADS score, mean [SD]	
Depression (n=260)	5.6 [4.0]
Anxiety (n=259)	5.2 [3.9]
HADS Depression score, n (%)	
1–7	194 (73.4%)
≥ 8	66 (25.0%)
Missing	4 (1.5%)
HADS Anxiety score, n (%)	
1–7	194 (73.4%)
≥ 8	65 (24.6%)
Missing	5 (1.9%)

#### Psychometric Testing of the CRED–V1

##### Descriptive Statistics

The greatest distress sources were “breathlessness” (Item 8) and “lack of control in future” (Item 19). For “oxygen therapy” (Item 3), “antibiotic/steroids” (Item 5) and “restriction in duties regarding work and volunteering” (Item 22)—40% or more reported no distress. Partly because “situation does not apply” and “no distress at all” were counted as “0”, most items’ distributions were skewed. For further details, see Table S2.

##### Exploratory Factor Analysis

For reflective constructs, the scree plot indicated a four-factor solution. Items 16 (missing collaboration with care team) and 20 (lack of physical training)—originally aligned to “treatment” (formative constructs)—were included in the analysis because they behaved similarly to items loading on “restriction in daily life”. Four others (items 15, 16, 33 and 34) were excluded for factor loadings <0.4. The Kaiser–Meyer–Olkin (KMO) value for the final factor analysis was 0.91, with 66.7% of the variance explained. The following factor pattern was revealed: The factor “unpredictability” (three items: 17–19) evolved, consistent with the initial conceptual framework. The developer-intended category “restriction in daily life” revealed three factors: “restricted mobility” (eight items: 20–27), “restricted relationships” (five items: 28–32) and “stigma” (two items: 35 and 36).

##### Path Model with Partial Least Squares–Structural Equation Modelling (PLS–SEM)

**Figure 4:**
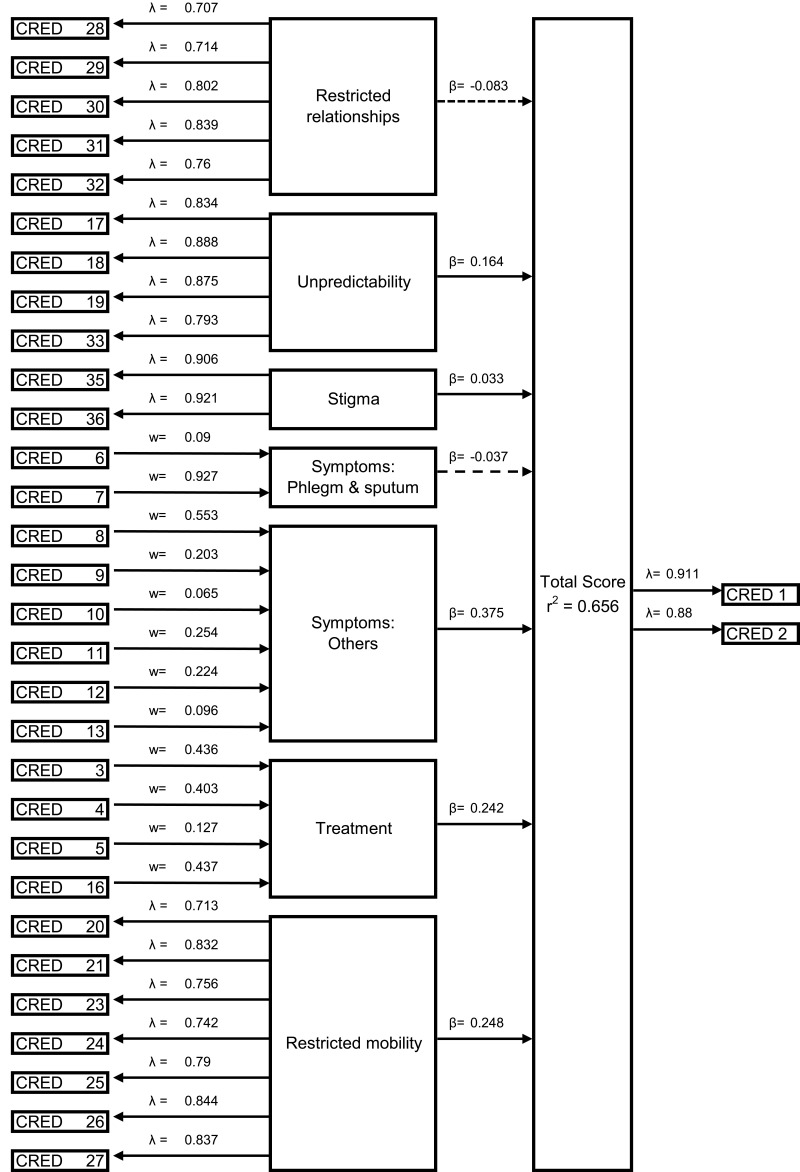
Path model (Figure Legend: λ = loading, w =weight, β = path coefficient, r2 = coefficient of determination).

After 4 items (items 15, 16, 22, 34) with loading values <0.7 were excluded, the final model included 32 items (for coefficients, see Figure 4).

##### Reflective Model Evaluation

The scale’s internal consistency was good (Cronbach’s alphas: 0.87 (unpredictability), 0.90 (restricted mobility), 0.82 (restricted relationships), 0.80 (stigma)). Convergent validity was good, with all four scales’ average variance extracted (AVE) >0.5 (restricted mobility: 0.63, restricted relationships: 0.59, unpredictability: 0.72, stigma: 0.83). The reflective constructs’ discriminant validity was also good: Fulfilling Fornell and Larcker’s criteria, each scale’s AVE root was higher than its correlation with other constructs, and all Heterotrait-Monotrait values <0.85.

##### Formative Model Evaluation

Treatment is a formative construct. As “Overall treatment distress” (Item 2) should reflect the four treatment items (Items 3–5, 16), it functions as an alternative measure of the same construct. Whereas, for the redundancy analysis model, each path coefficient should be >0.7, this item’s coefficient of 0.6 suggests that the “treatment” construct was inadequately covered by items 3–5 and 16. The range of variance inflation factors (VIFs) for all treatment and symptom items was 1.1–2.9, indicating no significant multicollinearity. The items’ weights and loadings were mostly significant, indicating adequate factor (construct) coverage. Three items (5, 7, 10) had non-significant weights, but significant loadings, indicating their inclusion. Only one item (14) had non-significant weight and loading, indicating its deletion. Symptoms were dichotomized as “Coughing” and “Phlegm” (Items 6, 7, respectively) versus all other symptoms (Items 8–13).

##### Structural Model Evaluation

VIFs were all <3, indicating no significant multicollinearity problems. Path coefficients were significant for “restricted mobility”, “unpredictability”, “symptoms” (other than “coughing” and “phlegm”), and “treatment”. The model’s R^2^ value was 0.656, indicating good explanatory value. For the formulae for calculating the CRED–TS, see Table 2.

**Table 2:** Formula to Calculate Subscores and Total Score

Subscores		
Treatment	=	((0.436 x Item 3) + (0.403 x Item 4) + (0.127 x Item 5) + (0.437 x Item 16)) / (0.436 + 0.403 + 0.127 + 0.437)
Symptoms cough and phlegm	=	((0.090 x Item 6) + (0.927 x Item 7) / (0.090 + 0.927)
Symptoms	=	((0.553 x Item 8) + (0.203 x Item 9) + (0.065 x Item 10) + (0.254 x Item 11) + (0.224 x Item 12) + (0.096 x Item 13)) / (0.553 + 0.203 + 0.065 + 0.254 + 0.224 + 0.096)
Unpredictability	=	((0.306 x Item 17) + (0.290 x Item 18) + (0.305 x Item 19) + (0.277 x Item 33)) / (0.306 + 0.290 + 0.305 + 0.277)
Restricted mobility	=	((0.168 x Item 20) + (0.208 x Item 21) + (0.173 x Item 23) + (0.169 x Item 24) + (0.170 x Item 25) + (0.191 x Item 26) + (0.185 x Item 27)) / (0.168 + 0.208 + 0.173 + 0.169 + 0.170 + 0.191 + 0.185)
Restricted relationships	=	((0.247 x Item 28) + (0.182 x Item 29) + (0.259 x Item 30) + (0.309 x Item 31) + (0.300 x Item 32)) / (0.247 + 0.182 + 0.259 + 0.309 + 0.300)
Stigma	=	((0.524 x Item 35) + (0.570 x Item 36)) / (0.524 + 0.570)
**Total Score**		
Total Score	=	(Treatment x 0.242) + (Symptoms cough and phlegm x −0.037) + (Symptoms x 0.375) + (Unpredictability x 0.164) + (Restricted mobility x 0.248) + (Restricted relationships x −0.083) + (Stigma x 0.033)

##### Construct Validity

Comparing the CRED–V1 with established questionnaires (HADS, CAT, mMRC) indicated that the regression model’s most important variable was the CAT score, followed by the HADS Depression score. The CRED–V1’s relationship with the HADS Anxiety score was non-linear: until a moderate anxiety level, the CRED and HADS anxiety scores correlated positively; however, after that, the CRED score plateaued (Table 3).

**Table 3:** Regression Model for CRED Total Score (CRED-TS)

	Estimate	Std. Error	t Value	Pr(>|t|)	
(Intercept)	0.0807921	0.1836378	0.440	0.660399	
HADS Anxiety	0.7566354	0.1625993	4.653	5.61e-06	***
I(HADS Anxiety^2)	−0.1965647	0.0756317	−2.599	0.009977	**
HADS Depression	0.4495528	0.0893554	5.031	1.01e-06	***
CAT	0.0373280	0.0064025	5.830	1.94e-08	***
mMRC 1	−0.0004071	0.1231735	−0.003	0.997366	
mMRC 2	0.2670244	0.1275182	2.094	0.037394	*
mMRC 3	0.4345974	0.1474147	2.948	0.003539	**
mMRC 4	0.6908214	0.1887968	3.659	0.000316	***
Sex	0.1807180	0.0677283	2.668	0.008187	**
FEV1 predicted %	−0.0042305	0.0017897	−2.364	0.018953	*

**Notes**: Signif. codes: 0 “***”, 0.001 “**”, 0.01 “*”.

## Discussion

The CRED–V1 PROM was developed following the relevant FDA Guideline. Guided by our model of illness-related emotional distress in respiratory disease,^7^ its conceptual framework was hypothesized and the preliminary 36-item list developed. Experts and patients confirmed the list’s content validity. In psychometric testing, the original conceptual framework was well reflected in the factor solution. Four items (14, 15, 22, 34) did not perform well and could not be included in the final path model. This indicated that these four items should be removed from the CRED total score. Cronbach’s alpha values were good for all reflective subscales.

Three of the four initial distress subcategories—’symptom distress’, “treatment distress”, “distress due to unpredictability”—matched the initial conceptual framework’s hypotheses. Psychometric testing indicated that the fourth, “restriction in daily life”, should be separated into “restricted relationships”, “restricted mobility” and “stigma”. Therefore, the psychometric testing represented our deductively derived framework^7^ and its application to Zanolari’s six subcategories, which evolved inductively from patient interviews.^8^ Of those subcategories, “stigma” was associated with the lowest emotional distress; however, this ranking might reflect social desirability bias because patients might be reluctant in revealing emotional distress in this area. For the CRED’s next draft, revision of bias-prone items will be considered.

The results regarding construct validity were as expected: The revised CRED–TS’s correlated positively with the CAT score, both HADS scores and the mMRC dyspnoea scale. Interestingly, the CAT score was the most important variable in the regression model, followed by the HADS depression score. While the CAT assesses a concept between COPD-specific and illness-specific restrictions in daily life (Figure 1), the HADS assesses symptoms related to anxiety and depression, that reflects overall level of psychological distress. Similarly, the CAF, which measures COPD-specific anxiety, showed a close relationship with the CAT and the HADS anxiety score.^33^ This indicates that the attempt to create an instrument that combines both – measuring emotional distress and COPD-specific problems – may have been successful.

Psychometric testing differentiated two symptoms clusters: “Cough” and “phlegm”, and the other symptoms. This was unexpected and mainly due to an unexpected pattern of the item “cough” that correlated negatively (though nonlinearly) with emotional distress in higher GOLD levels.

Our post-hoc analysis showed that patients with GOLD 1–3 experienced coughing as more distressing than those with GOLD 4. This is consistent with a previous study that reported more frequent and more severe coughing in moderate COPD compared to GOLD 4,^34^ and can be explained by the fact symptom distress correlates with symptom severity and frequency.^35^

This study has clear implications for practice and research. The CRED–V1 and revised versions will be used as PROMs to plan and evaluate COPD-related emotional distress reduction interventions. By addressing psychological distress regardless of whether it is pathological, the CRED–V1 targets the concept of COPD-based psychological suffering.^36^ Based on current evidence, successful psychological distress reduction interventions include components of cognitive-behavioural approaches, pulmonary rehabilitation, and self-management support.^36^,^37^ These will be addressed as part of further intervention development.

One major methodological strength of this questionnaire’s development was the consistent involvement of patients to ensure that the CRED–V1 assesses patient-relevant topics. One of the CRED–V1’s strengths is that its total score facilitates comparisons those of with other PROMs. However, despite these positive features, the questionnaire also needs further adaptions before implementation in clinical practice: The skewed response distribution indicates that “easier” items are needed, eg., items relevant to patients with mild COPD, who are less likely to give socially desirable responses. Also, the two formative constructs’ statistical results (“symptom distress” and “treatment distress”) raise the question of whether all relevant symptoms and treatments are included in the item list, and call for further distinctions between COPD- and other-illness-related emotional distress. Therefore, to distinguish between the two, further level-2 questions will be necessary. The scoring categories’ overlap calls for a revision of the 6-point Likert scales. To address these issues, following a revision and refinement of the current questionnaire, a second validation study is planned. The responsiveness of the scale has yet to be established and will be part of future research.

This study has some limitations: This single centre study only included German-speaking patients. Its transferability to other populations must be established in future. The cross-sectional design does not permit the establishment of causal relationship.

After that second validation, a translation of the instrument into English is planned.

## Conclusion

COPD-Related Emotional Distress (CRED) was operationalized as a patient-reported outcome measure (CRED–V1) with good content validity and good internal consistency. Construct validity was established via close relationships with CAT and HADS scores. Still, implementing the CRED–V1 into clinical practice will require further refinement.

## Abbreviations

CAT, COPD Assessment Test; COPD, Chronic Obstructive Pulmonary Disease; CRED, COPD-related emotional distress questionnaire; CRED-TS, COPD-related emotional distress questionnaire–total score; FDA, U.S. Food and Drug Administration; HADS, Hospital Anxiety and Depression Scale; mMRC, modified Medical Research Counsel; PROM, patient-reported outcome measure.
